# Recorded diagnosis of overweight/obesity in primary care is linked to obesity care performance rates

**DOI:** 10.1038/s41390-024-03619-0

**Published:** 2024-10-08

**Authors:** Shlomit Shalitin, Moshe Phillip, Michal Yackobovitch-Gavan

**Affiliations:** 1 The Jesse Z. and Sara Lea Shafer Institute of Endocrinology and Diabetes, National Center for Childhood Diabetes Schneider Children’s Medical Center of Israel, Petah Tikva, Israel; 2 Faculty of Medical and Health Sciences, Tel Aviv University, Tel Aviv, Israel; 3 Dept. of Epidemiology and Preventive Medicine, School of Public Health, Faculty of Medicine, Tel Aviv University, Tel Aviv, Israel

## Abstract

**Background:**

Periodical BMI measurement during visits with primary care pediatricians (PCP) can be central to diagnosing, managing, and treating overweight/obesity. The aim was to evaluate among children and adolescents with similar BMI percentiles, whether recording a formal diagnosis by PCP, of overweight/obesity is associated with improved performance rates of obesity-related care.

**Methods:**

The electronic database of the largest health maintenance organization in Israel was searched for all patients aged 2–18 years with BMI recorded at a visit with the PCP during 2017–2023. Among children with BMI percentiles consistent with overweight/obesity, performance rates of obesity care were compared between those with a recorded diagnosis of “overweight”/“obesity” placed by the PCP, and those with similar BMI percentiles without these recorded diagnoses.

**Results:**

Among children with versus without recorded diagnoses of overweight/ obesity, rates were higher of referrals for screening measurements for obesity-related comorbidities, for dietitian and endocrine counseling, of performing subsequent BMI measurements, and of prescribing anti-obesity medications (*p* < 0.001 for all). Obesity-related comorbidities were more prevalent among those with than without recorded diagnoses (*P* < 0.001).

**Conclusions:**

Beyond BMI measurement, a recorded diagnosis of overweight/obesity by a PCP is linked to higher rates of obesity care performance and interventions, which may improve clinical outcomes.

**Impact statement:**

- BMI measurement during visits with primary care pediatricians (PCP) can be central to diagnosing, managing, and treating overweight/obesity.
- We evaluated among children and adolescents with similar BMI percentiles, whether recording a formal diagnosis by PCP, of overweight/obesity is associated with improved performance rates of obesity-related care.
- We found that among children with versus without recorded diagnoses of overweight/obesity, rates were higher of referrals for screening measurements for obesity-related comorbidities, for dietitian and endocrine counseling, and of prescribing anti-obesity medications.
- Therefore, PCP should increase rates of recording diagnoses of overweight/obesity, to promote screening for obesity-related comorbidities, and aim to treat obesity as a chronic disease.

## Introduction

Obesity is a global public health challenge with worldwide increased incidence. Childhood obesity has reached pandemic proportions and is currently one of the most prevalent public health problems.^1^ The pathophysiology of excess weight gain is complex, and includes interactions between genetic, biological, behavioral (physical inactivity, increased screen time), and socioeconomic factors.^2–4^

The increasing prevalence of childhood obesity is associated with the emergence of comorbidities previously considered to be “adult” diseases, including hypertension, dyslipidemia, type 2 diabetes, obstructive sleep apnea (OSA), and non-alcoholic fatty liver disease (NAFLD).^5^ The negative health, social, and economic consequences of obesity can impact a child’s quality of life. Moreover, children with obesity are likely to become adults with obesity; the latter confers increased risks of morbidity and mortality.^6^ Therefore, controlling the childhood obesity pandemic has become a top public health priority worldwide.

In clinical practice, body mass index (BMI) frequently serves as both a screening and diagnostic tool for detecting excess body adiposity, because it is easy to use and inexpensive. For children and teens, BMI interpretation is age- and sex-specific, and a child’s BMI category is determined using an age-and sex-specific percentile for BMI. The World Health Organization (WHO) has defined age-specific cutoff values of BMI for pediatric overweight and obesity.^7,8^

Periodical BMI measurement during regular visits with primary care pediatricians (PCP) is central to diagnosing overweight and obesity, and to managing and tracking overweight and obesity in children and adolescents.

Early and accurate classifications of overweight and obesity, and identifying obesity-related comorbidities are fundamental to providing appropriate treatment. This is important because young patients and their parents often do not perceive overweight and obesity as health problems; and may thus not benefit from health behavior and lifestyle change treatment, or pharmacological anti-obesity medication treatment.

In the electronic files of Clalit Health Services (CHS), an Israeli payer-provider integrated healthcare system, the diagnosis of “overweight” and “obesity” is not automatically recorded based on patients’ BMI categories. Instead, it must be manually recorded in the patient’s file by the PCP.

The aim of this study was to evaluate among children and adolescents with similar BMI percentiles, whether recording a formal diagnosis of “overweight” or “obesity” by a PCP, is associated with improved performance rates of obesity-related care within the primary care setting. Specifically, we examined the relationship between the recording of an overweight or obesity diagnosis and the screening for obesity-related comorbidities, as well as the association between such documentation and the provision of clinical interventions (including referral for dietician or endocrine consultation or prescribing anti-obesity medications) and follow-up care. Those with a recorded diagnosis of “overweight” or “obesity” were compared to those without such documentation.

## Methods

This observational retrospective study was conducted using the electronic database of CHS, the largest health maintenance organization in Israel, serving about 54% of the national population. The database is accumulated by continuous real-time input from physicians and health service providers and includes patient demographic, socioeconomic, and clinical characteristics, hospital discharge and outpatient clinic diagnoses, laboratory test results, medical treatments, and medication dispensation information. Data were extracted from CHS using the Clalit Research Data sharing platform powered by MDClone (https://www.mdclone.com).

### Study population

CHS uses WHO growth charts for children aged 0-19 years. The electronic database of CHS was searched for all the patients aged 2-18 years with at least one BMI value that adhered to the WHO definitions of overweight or obesity at a visit with a PCP during 2017-2023. The independent binary variable was a recorded formal diagnosis of “overweight” or “obesity” according the International Statistical Classification of Diseases and Related Health Problems 10th Revision (ICD10 codes) placed by the PCP in the electronic medical chart at or immediately after the BMI measurement.

Children with BMI values that met the WHO criteria for “overweight” and “obesity” and had a recorded diagnosis of “overweight” or “obesity” (as indicated by ICD-10 codes) were compared to those with similar BMI values who did not have a documented diagnosis.

Study exclusion criteria were improbable measurements, for example, BMI > 60 kg/m^2^ or a difference in BMI measurements >5 between the first two measurements in an interval of 1–2 years.

Four comparison groups were established according to the first documented BMI value during the study period that adhered to the WHO definitions of overweight and obesity. Overweight was defined as 97th < BMI ≤ 99.9th percentile (2 < BMI-Z score ≤ 3) and 85th < BMI ≤ 97th percentile (1 < BMI-Z score ≤ 2) for ages 2–5 years and >5 to 18 years, respectively. Obesity was defined as BMI > 99.9th percentile (BMI-Z score > 3) and BMI > 97th percentile (BMI-Z score > 2) for the respective age ranges.

### Data measurements and variables

From the medical files, demographic parameters (sex, ethnicity, age) and socioeconomic position (SEP) were retrieved. Data collected during the study period included anthropometric parameters (height and weight as measured by nurses or PCP and calculated BMI), blood pressure measurements, and the presence of obesity-related comorbidities. The latter included dyslipidemia, type 2 diabetes, impaired glucose tolerance, hypertension, OSA, NAFLD, and pseudotumor cerebri; and in adolescent girls, polycystic ovaries. Data were also retrieved of screening performance during the study period for blood glucose, HbA1C, lipid profile, liver enzymes, liver ultrasound, and sleep studies. Further data included follow-up during the study period of additional weight and height measurements, referral to a dietitian or an endocrine evaluation, and the use of anti-obesity medications (orlistat, GLP-1 analogs, and metformin).

The SEP index of the Israel Central Bureau of Statistics classifies and characterizes statistical areas within municipalities and local councils based on the socioeconomic status of their populations. This index is derived from an adjusted calculation of 14 variables that assess social and economic levels in the domains of demographics, education, standard of living, and employment. The SEP classification for a given residence is determined according to the socioeconomic level of the population within towns and neighborhoods, as evaluated in 2015.^9^ The SEP clusters are scored on a scale from 1 to 10, with 1 indicating the lowest SEP and 10 representing the highest. For the purposes of this study, these 10 clusters were categorized into three groups: low SEP (clusters 1-4), medium SEP (clusters 5-6) and high SEP (clusters 7-10).

BMI was calculated as weight (in kilograms) divided by height (in meters) squared. To compare BMI values across age groups by sex, BMI-Z scores were calculated using the growth chart percentiles of the WHO (7,8).

The study was approved by the local institutional ethics committee of Rabin Medical Center (RMC-0606-23) in keeping with the principles of the Declaration of Helsinki.

Written subject informed consent was not required due to the retrospective design and the anonymous collection of the data.

### Statistical analysis

Statistical analyses were performed using SPSS software, version 29 (SPSS, Inc., Chicago, Illinois). Data are presented as *n* (%) or median (interquartile range for skewed distributions).

The four groups described above were compared for each sex, stratified to age group (2-5 years and >5-18 years), using the Pearson’s chi-square test for categorial variables and the Kruskal–Wallis 1-way ANOVA test for numerical variables with a skewed distribution. Multiple comparisons post-hoc analysis by the Bonferroni method was used to compare between pairs of groups.

Differences between males and females within each group were evaluated using Pearson’s chi-square test for categorial variables or the Mann–Whitney U-test for numerical variables with a skewed distribution. Bonferroni corrections for multiple comparisons were conducted for all the study outcomes.

For each age group, linear regression models were conducted for the association between a recorded diagnosis of overweight or obesity and a change in BMI-Z score in a 1–2-year interval from the first measurement. The models were adjusted for possible confounders. The dependent variable was a change in BMI-Z score in a 1–2-year interval. The independent variable was the four comparison groups. The model was adjusted for sex, age, baseline BMI-Z score, SEP, ethnicity, and a diagnosis of any obesity-related comorbidity.

## Results

**Fig. 1:**
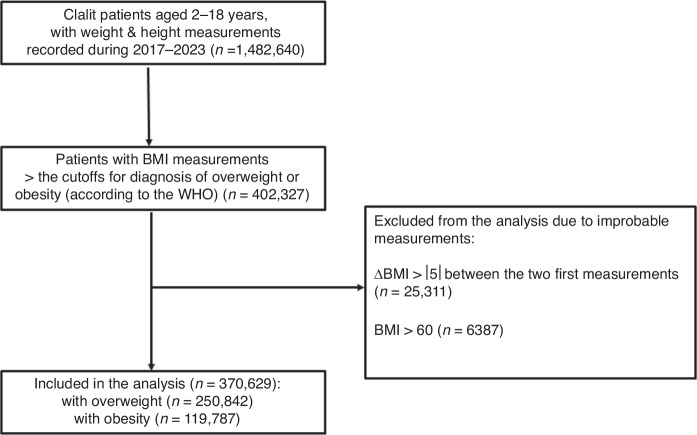
Flow chart of the individuals included in the study.

During the study period, 1,577,277 children (51.5% males) aged 2–18 years (median 6.9 years [interquartile range 4.3, 13.1]) were insured by CHS; 1,482,640 (94%) of them had at least one recorded BMI measurement, and 59% had two or more measurements. Of the entire sample, 402,327 (27.1%) had a BMI measurement above the cutoff for diagnosing overweight and obesity. After excluding from the analysis those with improbable measurements (6387 with a documented BMI > 60 kg/m^2^ and 25,311 with a difference in BMI measurements >5 between the first two documented measurements during an interval of 1–2 years), the final cohort included 370,629 children. Of them, 27,850 were aged 2–5 years, including 14,756 (53%) males; and 342,779 were aged >5–18 years, including 179,882 (52.5%) males (Fig. 1).

Among the younger group, 21,136 (75.9%) were defined as having overweight and 6714 (24.1%) as having obesity, according to the WHO criteria. Among the older group, the respective numbers were 229,706 (67%) and 113,073 (33%).

Formal diagnoses of “overweight” were not recorded for 9536 (45.1%) of the younger group and 112,604 (49%) of the older group. Formal diagnoses of “obesity” were not recorded for 2158 (32.1%) and 20,829 (18.4%) of the respective groups.

Table 1 presents characteristics of the study cohort according to the presence of overweight or obesity, and the recording of a formal diagnosis of overweight or obesity. For both age groups, and for both males and females, children with recorded diagnoses of overweight or obesity were older than those with similar BMI and without recorded diagnosis of overweight or obesity (*p* < 0.001).

**Table 1:** Characteristics of the study cohort according to the presence of overweight or obesity, and the recording of a formal diagnosis of overweight or obesity.

	Patients with overweight without a recorded diagnosis of overweight	Patients with overweight with a recorded diagnosis of overweight	Patients with obesity without a recorded diagnosis of obesity	Patients with obesity with a recorded diagnosis of obesity	P1
Age 2–5 years					
Total	9536 (34.2)	11,600 (41.7)	2158 (7.7)	4556 (16.4)	
Sex					
males	5121 (53.7)^a^	5901 (50.9)^b^	1254 (58.1)^c^	2480 (54.4)^d^	**<0.001**
females	4415 (46.3)	5699 (49.1)	904 (41.9)	2076 (45.6)	
Age, years					
males	2.7 (2.2, 3.8)^a^	3.3 (2.3, 4.4)^b^	3.3 (2.4, 4.4)^b^	3.8 (2.8, 4.6)^c^	**<0.001**
females	2.6 (2.2, 3.5)^a^	3.3 (2.3, 4.3)^b^	3.1 (2.4, 4.2)^b^	3.6 (2.7, 4.5)^c^	**<0.001**
P2	**<0.001**	0.633	**0.024**	**0.006**	
BMI-Z score (first measurement)					
males	2.30 (2.13, 2.55)^a^	2.33 (2.15, 2.59)^b^	3.55 (3.21, 4.43)^c^	3.65 (3.25, 4.33)^c^	**<0.001**
females	2.28 (2.12, 2.52)^a^	2.34 (2.15, 2.60)^b^	3.51 (3.22, 4.30)^c^	3.55 (3.22, 4.09)^c^	
P2	**<0.001**	0.307	0.63	**<0.001**	**<0.001**
Height-Z (first measurement)					
males	−0.35 (−0.28, 0.49)^a^	0.10 (−0.76, 0.94)^b^	−0.69 (−2.98, 0.65)^c^	0.66 (−0.32, 1.54)^d^	**<0.001**
females	−0.31 (−1.20, 0.48)^a^	0.17 (0.61, 0.97)^b^	−0.79 (−3.41, 0.65)^c^	0.59 (−0.22, 1.37)^d^	
P2	0.268	**<0.001**	0.179	0.235	**<0.001**
Ethnicity					
males					
Jews	4054 (79.2)^a^	4455 (75.5)^b^	778 (62.0)^c^	1711 (69.0)^d^	**<0.001**
Arabs	1067 (20.8)	1446 (24.5)	476 (38.0)	769 (31.0)	
females					
Jews	3566 (80.8)^a^	4371 (76.7)^b^	580 (64.2)^c^	1520 (73.2)^d^	**<0.001**
Arabs	849 (19.2)	1328 (23.3)	324 (35.8)	556 (26.8)	
P2	0.051	0.129	0.315	**0.002**	
SEP level					
males					
Low	1680 (35.2)^a^	2095 (38.4)^b^	558 (49.6)^c^	1065 (46.9)^d^	**<0.001**
Medium	1458 (30.6)	1738 (31.8)	348 (30.9)	219 (31.7)	
High	1628 (34.2)	1627 (29.8)	220 (19.5)	487 (21.4)	
females					
Low	1387 (33.2)^a^	1981 (37.4)^b^	400 (48.5)^c^	846 (43.9)^d^	**<0.001**
Medium	1355 (32.5)	1694 (32.0)	257 (31.2)	673 (35.0)	
High	1431 (34.3)	1618 (30.6)	167 (20.3)	406 (21.1)	
P2	0.077	0.553	0.887	0.065	
Age >5–18 years					
Total	11,2604 (32.9)	117,102 (34.2)	20,829 (6.1)	92,244 (26.9)	
Sex					
males	57,965 (51.5)^a^	57,534 (49.1)^b^	12,551 (60.3)^c^	51,832 (56.2)^d^	**<0.001**
females	54,639 (48.5)	59,568 (50.9)	8278 (39.7)	40,412 (43.8)	
Age, years					
males	11.6 (7.3,14.0)^a^	12.1 (8.3,14.2)^b^	9.7 (6.2,13.2)^c^	11.6 (8.7,14.0)^d^	**<0.001**
females	11.2 (7.3,13.8)^a^	11.8 (8.1,14.2)^b^	9.2 (6.0,13.1)^c^	11.5 (8.3,14.1)^b^	**<0.001**
P2	**<0.001**	**<0.001**	**<0.001**	**<0.001**	
BMI-Z score (first measurement)					
males	1.35 (1.17, 1.58)^a^	1.49 (1.26, 1.74)^b^	2.39 (2.16, 2.78)^c^	2.57 (2.26, 2.98)^d^	**<0.001**
females	1.32 (1.16, 1.56)^a^	1.48 (1.25, 1.72)^b^	2.33 (2.14, 2.66)^c^	2.48 (2.21, 2.85)^d^	**<0.001**
P2	**<0.001**	**<0.001**	**<0.001**	**<0.001**	
Height-Z (first measurement)					
males	0.11 (−0.65, 0.83)^a^	0.23 (−0.50, 0.96)^b^	0.37 (−0.55, 1.20)^c^	0.56 (−0.17, 1.31)^d^	**<0.001**
females	0.07 (− 0.64, 0.77)^a^	0.17 (−0.52, 0.86)^b^	0.19 (−0.68, 1.03)^b^	0.43 (−0.28, 1.15)^c^	**<0.001**
P2	**<0.001**	**<0.001**	**<0.001**	**<0.001**	
Ethnicity					
males					
Jews	38,817 (67.0)^a^	39,376 (68.4)^b^	7,133 (56.8)^c^	32,766 (63.2)^d^	
Arabs	19,148 (33.0)	18,158 (31.6)	5,418 (43.2)	19,066 (36.8)	**<0.001**
females					
Jews	37,210 (68.1)^a^	41,795 (70.2)^b^	4853 (58.6)^c^	26,779 (66.3)^d^	
Arabs	17,429 (31.9)	17,773 (29.8)	3425 (41.4)	13,633 (33.7)	**<0.001**
P2	**<0.001**	**<0.001**	**0.01**	**<0.001**	
SEP level					
males					
Low	22,508 (41.9)^a^	22,672 (42.4)^a^	5824 (50.3)^b^	22,620 (47.0)^c^	
Medium	14,806 (27.6)	15,978 (29.9)	3047 (26.3)	14,535 (30.3)	**<0.001**
High	16,408 (30.5)	14,807 (27.7)	2718 (23.5)	10,935 (22.7)	
females					
Low	20,754 (41.0)^a^	22,723 (41.0)^a^	3744 (49.3)^b^	17,092 (45.5)^c^	
Medium	14,115 (27.9)	16,926 (30.6)	2166 (27.8)	11,645 (31.0)	**<0.001**
High	15,748 (31.1)	15,719 (28.4)	1738 (22.9)	8797 (23.5)	
P2	**0.012**	**<0.001**	0.058	**<0.001**	

The data are presented as *n* (%) or median (interquartile range, skewed distribution).

The bold values in Table 1 was considered statistically significant for P1 < 0.05 and P2 < 0.05.

P1 represents *P* values for the difference between the four comparison groups using the Pearson chi-square test for categorial variables or the Kruskal–Wallis 1-way ANOVA test for numerical variables with a skewed distribution. Multiple comparisons post-hoc analysis by the Bonferroni method was used to compare between each pair of groups. Rates with different superscripts (a, b, c, d) differ significantly from each other. P2 represents the *P* value for the differences between males and females within each group using the Pearson chi-square test for categorial variables or the Mann–Whitney *U*-test for numerical variables with a skewed distribution.

In the younger group, the median BMI-Z score was significantly higher among those with than without a recorded diagnosis of overweight (*p* < 0.001) and was not significantly different between those with and without a recorded diagnosis of obesity. Median BMI-Z scores were lower among females with overweight without a recorded diagnosis and females with obesity with a recorded diagnosis of obesity, compared to their male counterparts (*p* < 0.001). The difference in standard deviation (SD) was about 0.1. In the older group, the median BMI-Z score was significantly higher among those with than without a recorded diagnosis of overweight or obesity (*p* < 0.001). Median BMI-Z scores were lower among females than males in all four comparison groups (<0.1 SD difference). In both age groups, height-Z score was significantly higher among those with than without a recorded diagnosis of overweight or obesity (*p* < 0.001). The difference was the most substantial in the younger group of children with obesity.

In both age groups and both sexes, a higher proportion of children with obesity from Jewish than from Arab origin had a recorded diagnosis of obesity (*P* < 0.001). In both age groups and both sexes, SEP was higher among children with than without a recorded diagnosis of obesity (*P* < 0.001).

Table 2 presents the documented obesity-related comorbidities according to the presence of overweight or obesity, and according to the recording of a formal diagnosis of overweight or obesity. In the younger group, the rates of recorded obesity-related comorbidities were low. For both males and females with obesity, recorded diagnoses of dyslipidemia, OSA, and NAFLD were more prevalent among those with than without a recorded diagnosis (*P* < 0.001 for all the comparisons). Also for the overweight groups, recorded diagnoses of NAFLD were more prevalent among those with than without a recorded diagnosis (*P* < 0.001).

**Table 2:** Documented obesity-related comorbidities according to the presence of overweight or obesity, and the recorded diagnosis of overweight or obesity.

	Patients with overweight without a recorded diagnosis of overweight	Patients with overweight with a recorded diagnosis of overweight	Patients with obesity without a recorded diagnosis of obesity	Patients with obesity with a recorded diagnosis of obesity	P1
Age 2–5 years					
Total	9536 (34.2)	11,600 (41.7)	2158 (7.7)	4556 (16.4)	
Sex					
males	5121 (53.7)^a^	5901 (50.9)^b^	1254 (58.1)^c^	2480 (54.4)^d^	<**0.001**
females	4415 (46.3)	5699 (49.1)	904 (41.9)	2076 (45.6)	
Dyslipidemia					
males	10 (0.2)^a^	21 (0.4)^a^	4 (0.3)^a^	26 (1.0)^b^	<**0.001**
females	9 (0.2)^a^	29 (0.5)^b^	5 (0.6)^b^	25 (1.2)^c^	<**0.001**
P2	0.925	0.265	0.621	0.721	
IGT					
males	1 (0.0)	2 (0.0)	0	3 (0.1)	0.166
females	0	1 (0.0)	0	0	0.73
P2	0.999	0.999		0.315	
Hypertension					
males	3 (0.1)^a^	6 (0.1)^ab^	3 (0.2)^ab^	15 (0.6)^b^	< **0.001**
females	0^a^	9 (0.2)^b^	3 (0.3)^bc^	10 (0.5)^c^	< **0.001**
P2	0.303	0.599	0.687	0.72	
OSA					
males	27 (0.5)^a^	55 (0.9)^b^	8 (0.6)^ab^	57 (2.3)^c^	**<0.001**
females	32 (0.7)^a^	58 (1.0)^a^	8 (0.9)^a^	41 (2.0)^b^	<**0.001**
P2	0.273	0.708	0.685	0.518	
NAFLD					
males	0^a^	24 (0.4)^b^	0^a^	42 (1.7)^c^	**<0.001**
females	2 (0.0)^a^	42 (0.7)^b^	0^a^	51 (2.5)^c^	<**0.001**
P2	0.416	0.025		0.087	
PTC					
males	3 (0.1)^a^	2 (0.0)^a^	1 (0.1)^a^	6 (0.2)^b^	0.02
females	1 (0.0)	2 (0.0)	0	0	0.789
P2	0.724	0.999	0.972	0.067	
Age > 5–18 years					
Total	112,604 (32.9)	117,102 (34.2)	20,829 (6.1)	92,244 (26.9)	
Sex					
males	57,965 (51.5)^a^	57,534 (49.1)^b^	12,551 (60.3)^c^	51,832 (56.2)^d^	<**0.001**
females	54,639 (48.5)	59,568 (50.9)	8278 (39.7)	40,412 (43.8)	
Dyslipidemia					
males	245 (0.4)^a^	671 (1.2)^b^	45 (0.4)^a^	1095 (2.1)^c^	<**0.001**
females	419 (0.8)^a^	1067 (1.8)^b^	41 (0.5)^c^	1030 (2.5)^d^	<**0.001**
P2	**<0.001**	**<0.001**	0.163	**<0.001**	
Type 2 diabetes					
males	7 (0.0)^a^	21 (0.0)^a^	4 (0.0)^a^	79 (0.2)^b^	**<0.001**
females	12 (0.0)^a^	30 (0.1)^b^	3 (0.0)^a^	89 (0.2)^c^	<**0.001**
P2	0.295	0.319	0.999	0.188	
IGT					
males	11 (0.0)^a^	49 (0.1)^b^	0^a^	123 (0.2)^c^	**<0.001**
females	16 (0.0)^a^	46 (0.1)^b^	2 (0.0)^a^	101 (0.2)^c^	<**0.001**
P2	0.356	0.708	0.308	0.752	
Hypertension					
males	168 (0.3)^a^	442 (0.8)^b^	27 (0.2)^a^	923 (1.8)^c^	<**0.001**
females	77 (0.1)^a^	232 (0.4)^b^	20 (0.2)^c^	490 (1.2)^d^	<**0.001**
P2	**<0.001**	**<0.001**	0.806	**<0.001**	
OSA					
males	59 (0.1)^a^	102 (0.2)^b^	25 (0.2)^b^	183 (0.4)^c^	**<0.001**
females	50 (0.1)^a^	136 (0.2)^b^	18 (0.2)^b^	146 (0.4)^c^	<**0.001**
P2	0.647	0.061	0.898	0.879	
NAFLD					
males	95 (0.2)^a^	535 (0.9)^b^	31 (0.2)^a^	1636 (3.2)^c^	**<0.001**
females	90 (0.2)^a^	450 (0.8)^b^	12 (0.1)^a^	1232 (3.0)^c^	<**0.001**
P2	0.973	0.002	0.152	0.359	
PTC					
males	13 (0.0)^a^	18 (0.0)^a^	2 (0.0)^a^	83 (0.2)^b^	**<0.001**
females	31 (0.1)^a^	105 (0.2)^b^	4 (0.1)^a^	205 (0.5)^c^	<**0.001**
P2	0.004	**<0.001**	0.352	**<0.001**	
PCO					
females	10 (0.0)^a^	27 (0.0)^b^	1 (0.0)^a^	50 (0.1)^c^	**<0.001**

The data are presented as *n* (%).

The bold values in Table 2 was considered statistically significant for P1 < 0.006 and P2 < 0.002.

P1 represents *P* values for the differences between the four comparison groups using the Pearson chi-square test or Fisher’s exact test. P1 < 0.006 is considered significant after the Bonferroni correction (8 comorbidities). Multiple comparisons post-hoc analysis by the Bonferroni method was used to compare between each pair of groups. Rates with different superscripts (a, b, c, d) differ significantly from each other.

P2 represents *P* values for the differences between males and females within each group using the Pearson chi-square test or Fisher’s exact test. P2 < 0.002 was considered significant after the Bonferroni correction (7 comorbidities × 4 groups, 28 comparisons).

*IGT* Impaired glucose tolerance, *OSA* obstructive sleep apnea, *NAFLD* non-alcoholic fatty liver disease, *PTC* pseudotumor cerebri, *PCO* polycystic ovary.

In both sexes of the older age group, the rates of recorded diagnoses of dyslipidemia, impaired glucose tolerance, hypertension, OSA and NAFLD, and polycystic ovaries were higher among children with than without recorded diagnoses (*P* < 0.001 for all the comparisons). Among children with obesity, the rates of recorded diagnoses of type 2 diabetes and of pseudotumor cerebri were higher among those with than without a recorded diagnosis (*P* < 0.001 for both comparisons).

In all the comparison groups (except of children with obesity without a recorded formal diagnosis), recorded diagnoses of dyslipidemia and pseudotumor cerebri were more prevalent in females than males; and of hypertension, less prevalent. For all these comparisons, *P* < 0.001.

Table 3 presents rates of referral for evaluating obesity-related comorbidities, dietitian and endocrine evaluations, prescriptions of anti-obesity medications, and changes in BMI-Z scores during a 1–2-year interval, according to the presence of overweight or obesity, and to the recording of a formal diagnosis of overweight or obesity. In both age groups and both sexes, for children with overweight and with obesity, the rates of referral to several evaluations were higher among children with than without recorded diagnoses of overweight or obesity (*P* < 0.001 for all the comparisons). The evaluations considered here were referrals to blood pressure measurement, blood and imaging tests for screening for metabolic obesity-related comorbidities (blood glucose, HbA1c, liver enzymes, lipid profile, and liver ultrasound), and sleep studies.

**Table 3:** Referrals for evaluation for obesity-related comorbidities, dietitian and endocrine counseling, prescriptions of anti-obesity medications and change in BMI-Z scores during a 1–2-year interval according to the presence of overweight or obesity and the recorded diagnosis of overweight or obesity.

	Patients with overweight without a recorded diagnosis of overweight	Patients with overweight with a	Patients with obesity without a recorded diagnosis of obesity	Patients with obesity with a	P1
		recorded diagnosis of overweight		recorded diagnosis of obesity	
Age 2–5 years					
Total	9536 (34.2)	11,600 (41.7)	2158 (7.7)	4556 (16.4)	
Sex					
males	5121 (53.7)^a^	5901 (50.9)^b^	1254 (58.1)^c^	2480 (54.4)^d^	**<0.001**
females	4415 (46.3)	5699 (49.1)	904 (41.9)	2076 (45.6)	
Blood pressure measurement					
males	456 (8.9)^a^	2011 (34.1)^b^	131 (10.4)^a^	1026 (41.4)^c^	**<0.001**
females	332 (7.5)^a^	1934 (33.9)^b^	90 (10.0)^c^	821 (39.5)^d^	**<0.001**
P2	0.016	0.889	0.765	0.223	
Referral to blood glucose measurement					
males	1632 (31.9)^a^	2915 (49.4)^b^	410 (32.7)^a^	1512 (61.0)^c^	**<0.001**
females	1337 (30.3)^a^	3038 (53.3)^b^	310 (34.3)^c^	1318 (63.5)^d^	**<0.001**
P2	0.1	**<0.001**	0.465	0.086	
Referral to HbA1C measurement					
males	198 (3.9)^a^	1166 (19.8)^b^	65 (5.2)^c^	915 (36.9)^d^	**<0.001**
females	198 (4.5)^a^	1503 (26.4)^b^	57 (6.3)^c^	883 (42.5)^d^	**<0.001**
P2	0.145	**<0.001**	0.308	**<0.001**	
Referral to liver enzyme measurement					
males	1828 (35.7)^a^	3144 (53.3)^b^	456 (36.4)^a^	1594 (64.3)^c^	**<0.001**
females	1484 (33.6)^a^	3228 (56.6)^b^	332 (36.7)^a^	1367 (65.8)^c^	**<0.001**
P2	0.035	**<0.001**	0.899	0.281	
Referral to lipid profile measurement					
males	984 (19.2)^a^	2151 (36.5)^b^	261 (20.8)^a^	1318 (53.1)^c^	**<0.001**
females	849 (19.2)^a^	2469 (43.3)^b^	229 (25.3)^c^	1179 (56.8)^d^	**<0.001**
P2	0.895	**<0.001**	0.016	0.015	
Referral to liver ultrasound					
males	165 (3.2)^a^	383 (6.5)^b^	45 (3.6)^a^	244 (9.8)^c^	**<0.001**
females	174 (3.9)^a^	553 (9.7)^b^	40 (4.4)^a^	274 (13.2)^c^	**<0.001**
P2	0.066	**<0.001**	0.383	**<0.001**	
Referral to sleep study					
males	52 (1.0)^a^	90 (1.5)^b^	17 (1.4)^ab^	79 (3.2)^c^	**<0.001**
females	34 (0.8)^a^	64 (1.1)^a^	6 (0.7)^a^	56 (2.7)^b^	**<0.001**
P2	0.248	0.07	0.183	0.379	
Referral to a dietitian					
males	132 (2.6)^a^	677 (11.5)^b^	50 (4.0)^c^	669 (27.0)^d^	**<0.001**
females	140 (3.2)^a^	1063 (18.7)^b^	55 (6.1)^c^	759 (36.6)^d^	**<0.001**
P2	0.094	**<0.001**	0.033	**<0.001**	
Referral to an endocrinologist					
males	191 (3.7)^a^	350 (5.9)^b^	30 (2.4)^c^	282 (11.4)^d^	**<0.001**
females	169 (3.8)^a^	516 (9.1)^b^	23 (2.5)^c^	336 (16.2)^d^	**<0.001**
P2	0.844	**<0.001**	0.933	**<0.001**	
Additional BMI documentation					
males	3429 (67.0)^a^	5081 (86.1)^b^	716 (57.1)^c^	2153 (86.8)^d^	**<0.001**
females	2875 (65.1)^a^	4969 (87.2)^b^	497 (55.0)^c^	1759 (84.7)^d^	**<0.001**
P2	0.061	0.091	0.35	0.049	
1–2 years change in BMI-Z					
males	(*n* = 1478) −0.99 ^a^ (−1.66, −0.37)	(*n* = 2751) −0.38 ^b^ (−1.16, 0.38)	(*n* = 281) −1.59 ^c^ (-2.89, -0.56)	(*n* = 1161) −0.20 ^b^ (−1.02, 0.49)	**<0.001**
females	(*n* = 1194) −0.97 ^a^ (−1.62, −0.32)	(*n* = 2727) −0.30 ^b^ (−0.89, 0.30)	(*n* = 217) −1.32 ^c^ (−2.36, −0.44)	(*n* = 975) −0.32 ^b^ (−0.99, 0.24)	**<0.001**
P2	0.443	0.016	0.105	0.002	
Age >5-18 years					
Total	112,604 (32.9)	117,102 (34.2)	20,829 (6.1)	92,244 (26.9)	
Sex					
males	57,965 (51.5)^a^	57,534 (49.1)^b^	12,551 (60.3)^c^	51,832 (56.2)^d^	**<0.001**
females	54,639 (48.5)	59,568 (50.9)	8278 (39.7)	40,412 (43.8)	
Blood pressure measurement					
males	24,316 (41.9)^a^	36,748 (63.9)^b^	3552 (28.3)^c^	34,578 (66.7)^d^	**<0.001**
females	22,054 (40.4)^a^	37,836 (63.5)^b^	2131 (25.7)^c^	26,882 (66.5)^d^	**<0.001**
P2	**<0.001**	0.209	**<0.001**	0.545	
Referral to blood glucose measurement					
males	27,699 (47.8)^a^	37,300 (64.8)^b^	5140 (41.0)^c^	38,362 (73.9)^d^	**<0.001**
females	31,316 (57.3)^a^	44,383 (74.5)^b^	3930 (47.5)^c^	32,799 (81.2)^d^	**<0.001**
P2	**<0.001**	**<0.001**	**<0.001**	**<0.001**	
Referral to HbA1C measurement					
males	7793 (13.4)^a^	19,536 (34.0)^b^	1471 (11.7)^c^	27,212 (52.5)^d^	**<0.001**
females	9408 (17.2)^a^	25,041 (42.0)^b^	1233 (14.9)^c^	24,079 (59.6)^d^	**<0.001**
P2	**<0.001**	**<0.001**	**<0.001**	**<0.001**	
Referral to liver enzyme measurement					
males	29,342 (50.6)^a^	38,891 (67.6)^b^	5478 (43.6)^c^	3887 (75.0)^d^	**<0.001**
females	32,748 (59.9)^a^	45,658 (76.6)^b^	4128 (49.9)^c^	33,133 (82.0)^d^	**<0.001**
P2	**<0.001**	**<0.001**	**<0.001**	**<0.001**	
Referral to lipid profile measurement					
males	22,931 (39.6)^a^	33,804 (58.8)^b^	4268 (34.0)^c^	36,625 (70.7)^d^	**<0.001**
females	27,027 (49.5)^a^	41,132 (69.1)^b^	3382 (40.9)^c^	31,806 (78.7)^d^	**<0.001**
P2	**<0.001**	**<0.001**	**<0.001**	**<0.001**	
Referral to liver ultrasound					
males	2513 (4.3)^a^	4420 (7.7)^b^	411 (3.3)^c^	6545 (12.6)^d^	**<0.001**
females	3633 (6.6)^a^	7060 (11.9)^b^	400 (4.8)^c^	7268 (18.0)^d^	**<0.001**
P2	**<0.001**	**<0.001**	**<0.001**	**<0.001**	
Referral to sleep study					
males	146 (0.3)^a^	283 (0.5)^b^	37 (0.3)^a^	404 (0.8)^c^	**<0.001**
females	129 (0.2)^a^	212 (0.4)^b^	19 (0.2)^ab^	306 (0.8)^c^	**<0.001**
P2	0.634	0.002	0.451	0.73	
Referral to a dietitian					
males	2346 (4.0)^a^	6710 (11.7)^b^	900 (7.2)^c^	14,698 (28.4)^d^	**<0.001**
females	4672 (8.6)^a^	12,596 (21.1)^b^	992 (12.0)^c^	16,360 (40.5)^d^	**<0.001**
P2	**<0.001**	**<0.001**	**<0.001**	**<0.001**	
Referral to an endocrinologist					
males	2530 (4.4)^a^	3795 (6.6)^b^	292 (2.3)^c^	4848 (9.4)^d^	**<0.001**
females	3647 (6.7)^a^	5735 (9.6)^b^	273 (3.3)^c^	5641 (14.0)^d^	**<0.001**
P2	**<0.001**	**<0.001**	**<0.001**	**<0.001**	
Additional BMI documentation					
males	32,768 (56.5)^a^	45,602 (79.3)^b^	5098 (40.6)^c^	41,540 (80.1)^d^	**<0.001**
females	30,977 (56.7)^a^	47,632 (80.0)^b^	3212 (38.8)^c^	32,906 (81.4)^d^	**<0.001**
P2	0.585	0.003	0.009	**<0.001**	
Prescription of metformin					
males	13 (0.0)^a^	90 (0.2)^b^	12 (0.1)^b^	472 (0.9)^c^	**<0.001**
females	41 (0.1)^a^	214 (0.4)^b^	11 (0.1)^a^	711 (1.8)^c^	**<0.001**
P2	**<0.001**	**<0.001**	0.426	**<0.001**	
Prescription of orlistat					
males	2 (0.0)^a^	12 (0.0)^b^	0^a^	33 (0.1)^c^	**<0.001**
females	5 (0.0)^a^	42 (0.1)^b^	0^a^	63 (0.2)^c^	**<0.001**
P2	0.404	**<0.001**		**<0.001**	
Prescription of GLP1 analogs					
males	3 (0.0)^a^	132 (0.2)^b^	3 (0.0)^a^	818 (1.6)^c^	**<0.001**
females	14 (0.0)^a^	567 (1.0)^b^	2 (0.0)^a^	1427 (3.5)^c^	**<0.001**
P2	0.011	**<0.001**	1	**<0.001**	
1–2-year change in BMI-Z					
males	(*n* = 12,589) −0.30 ^a^ (−0.73, 0.08)	(*n* = 21,614) −0.04 ^b^ (−0.45, 0.34)	(*n* = 1781) −0.33 ^c^ (−0.89, 0.02)	(*n* = 19,933) −0.01 ^d^ (−0.42, 0.16)	**<0.001**
females	(*n* = 11,830) -0.22 ^a^ (−0.58, 0.09)	(*n* = 22,413) −0.01 ^b^ (−0.35, 0.33)	(*n* = 1035) −0.30 ^c^ (−0.75, 0.02)	(*n* = 15,379) −0.06 ^d^ (−0.34, 0.17)	**<0.001**
P2	**<0.001**	**<0.001**	0.083	**<0.001**	

The data are presented as *n* (%) or median (interquartile range, skewed distribution).

The bold values in Table 3 was considered statistically significant for P1 < 0.005 and P2 < 0.001.

P1 represents *P* values for the differences between the four comparison groups using the Pearson chi-square test or Fisher’s exact test for categorial variables or the Kruskal–Wallis 1-way ANOVA test for numerical variables with a skewed distribution. P1 < 0.005 was considered significant after the Bonferroni correction (11 distinct variables). Multiple comparison post-hoc analysis by the Bonferroni method was used to compare between each pair of groups. Rates with different superscripts (a, b, c, d) differ significantly from each other.

P2 represents *P* values for the differences between males and females within each group using the Pearson chi-square test or Fisher’s exact test for categorial variables or the Mann–Whitney *U*-test for numerical variables with a skewed distribution. P2 < 0.001 was considered significant after the Bonferroni correction (11 distinct variables × 4 groups, 44 comparisons).

Among children with overweight and obesity, the rates of referral to professional medical consultation (dietitian, pediatric endocrinologist) were higher among those with than without recorded diagnosis (*P* < 0.001 for all the comparisons). For both body weight categories, for those with compared to those without recorded diagnoses, the proportions were higher of children with additional documented BMI measurements (*P* < 0.001). Among females, the rates were higher of referral to evaluation tests for obesity-related comorbidities and referrals to professional medical consultation regarding most of the variables examined. Only in the older group, with obesity and with a recorded diagnosis, the proportion with an additional documented BMI measurement was higher among females than males (*P* < 0.001).

In the older group, for both sexes, the rates were higher for prescriptions of anti-obesity medications (metformin, orlistat, and GLP1 analogs) among children with than without recorded formal diagnoses of both overweight and obesity (*P* < 0.001 for all the comparisons). Among the children with recorded diagnoses of overweight and obesity, the rates of prescriptions of anti-obesity medications were higher among females than males (*P* < 0.001 for all the comparisons).

We compared changes in BMI-Z scores among the children with recorded additional measurements of BMI in an interval of 1-2 years. Accordingly, among those with both overweight and obesity, for both age groups and both sexes, the decrease in BMI-Z score was significantly less among those with than without a recorded diagnosis of overweight or obesity. These results were consistent in both age groups also after adjustment to possible confounders using a linear regression model that included patient sex, age, baseline BMI-Z score, SEP, ethnicity, and a diagnosis of any obesity-related comorbidity (Table 4).

**Table 4:** Linear regression model for the association between a diagnosis of overweight or obesity and a change in BMI-Z score in a 1–2 year interval, adjusted for possible confounders.

	B (SE)	*P*
Age 2–5 years		
Patients with overweight without a recorded diagnosis of overweight	−1.10 (0.05)	**<0.001**
Patients with overweight with a recorded diagnosis of overweight	−0.50 (0.05)	**<0.001**
Patients with obesity without a recorded diagnosis of obesity	−1.30 (0.06)	**<0.001**
Patients with obesity with a recorded diagnosis of obesity (Reference group)	0	
Age > 5–18 years		
Patients with overweight without a recorded diagnosis of overweight	−0.39 (0.01)	**<0.001**
Patients with overweight with a recorded diagnosis of overweight	−0.10 (0.01)	**<0.001**
Patients with obesity without a recorded diagnosis of obesity	−0.43 (0.01)	**<0.001**
Patients with obesity with a recorded diagnosis of obesity (Reference group)	0	

The dependent variable was a change in BMI-Z score in a 1–2-year interval. The independent variable was the four comparison groups (the presence of overweight or obesity, and a recorded diagnosis of overweight or obesity). The reference group was patients with obesity with a recorded diagnosis. The model was adjusted for sex, age, baseline BMI-Z score, socioeconomic position, ethnicity, and a diagnosis of any obesity-related comorbidity.

The bold values in Table 4 was considered statistically significant for *P* < 0.05.

## Discussion

In our large cohort, we observed that children and adolescents with a formal diagnosis of overweight or obesity recorded by the PCP were more likely to be referred for screening measurements for obesity-related comorbidities, for dietitian counseling or endocrine evaluation, and for performing subsequent BMI measurements, compared to those without a recorded diagnosis of overweight or obesity. For the >5–18 year-age group, among those with versus without recorded diagnoses of overweight or obesity anti-obesity medications were more commonly prescribed.

Placing a formal diagnosis of overweight or obesity indicates recognition of these diagnoses as a chronic condition by the PCP and patient. Although guidelines exist for obesity management by the PCP, the lack of established records of formal diagnoses of overweight or obesity may hamper evaluating screening performance of obesity-related comorbidities and referrals for obesity-treatment programs and their success. Indeed, ample evidence has been reported of suboptimal clinical documentation by PCP of diagnoses and management plans in medical records.^10–13^

The recommendations of the Israeli Health Ministry are measurements of height and weight at least once at age 2–4 years, at least once at age 5–7 years, and at least once in 3 years in children older than 14 years (https://israelhealthindicators.org/Measures/70/43). The vast majority (94%) of the children assessed for inclusion in our cohort had at least one recorded BMI measurement during the study period. However, among those with BMI measurements defined as overweight, 45.1% of those aged 2–5 years and 49% of those aged >5–18 years did not have a recorded formal diagnosis of “overweight”. Moreover, 32.1% of the children aged 2–5 years and 18.4% of those aged >5–18 years with BMI measurements defined as obesity did not have a recorded formal diagnosis of “obesity”. Notably, the median age was older among those who had recorded diagnoses of overweight or obesity than among those who did not.

Interestingly, both among children aged 2–5 years and >5–18 years, we report a male predominance for those who did not have recorded diagnoses of overweight and obesity. Of those who did not have recorded diagnoses, we also report a predominance of Arab compared to Jewish ethnicity among children aged 2–5 years with obesity, and children aged >5–18 years, both with overweight and obesity. Ram et al. previously reported higher rates of obesity among Arab than Jewish children aged 6–12 years, especially boys.^14^ This suggests that both families and PCPs in Arab communities may be less aware of the presence and implications of overweight and obesity in children. This may be related to sociocultural beliefs related to ideal body image, and an acceptance of larger body size.

In both sexes and age groups examined, SEP was higher among children with than without a recorded formal diagnosis of obesity. Lower SEP was reported to be associated with a higher prevalence of obesity in Israel.^15^ Thus, we can speculate that obesity is underdiagnosed or may be neglected in the lower SEP level, with decreased recording of a formal diagnosis.

Suboptimal documentation of obesity at PCP visits is a significant obstacle in management.^10,11^ In the national hospital and ambulatory medical care survey of 1155 pediatric patients with obesity, only 18% of the clinical notes showed accurate documentation of obesity.^16^ Importantly, those with compared to those without a documented diagnosis of obesity were significantly more likely to have a management plan in their notes.

Caregivers, pediatricians, and other pediatric healthcare providers can be slow to recognize abnormal weight status, even in the presence of severe obesity.^17^

Early and accurate classification of overweight and obesity using BMI measurements, identifying children and adolescents at high risk, and addressing obesity-related comorbidities are fundamental to providing appropriate treatment early in life. Among the children in our cohort aged >5-18 years, for those with a recorded formal diagnosis of overweight or obesity compared to those without, the rate was higher of diagnosed obesity-related comorbidities. This is probably due to lesser screening of these comorbidities among those without recorded diagnoses. Indeed, we report among children with compared to those without recorded diagnoses of both overweight and obesity, higher rates of referral for screening measurements for obesity-related comorbidities. Similarly, Hidirsah et al. ^13^ reported that documentation of an obesity diagnosis was associated with improved screening for comorbidities and prescriptions of anti-obesity medications among pediatric patients.

The 2023 expert committee of the American Academy of Pediatrics on child obesity recommended laboratory evaluations for children with obesity, for lipid abnormalities, abnormal glucose metabolism, and abnormal liver function, starting at age 10 years.^18^ This entails obtaining a fasting lipid panel, fasting glucose, and alanine transaminase and aspartate transaminase levels every 2 years. For children with overweight, the recommendation was only for a fasting lipid panel.^19^ For children aged 2–9 years with obesity, evaluation for lipid abnormalities may be considered.^20^ In children younger than 10 years, obtaining tests for abnormal glucose metabolism or liver function is not universally recommended, due to the lower risk profile for NAFLD and diabetes mellitus (especially in the absence of severe obesity). For evaluation of type 2 diabetes mellitus, additional risk factors need be considered, including family history, a history of gestational diabetes, signs of insulin resistance, and the use of obesogenic psychotropic medication. For NAFLD, additional risk factors include a family history of NAFLD, central adiposity, signs of insulin resistance, prediabetes or diabetes mellitus, dyslipidemia, and sleep apnea.^21^ Therefore, the relatively low referral rate for screening for obesity-related comorbidities in the younger group of our cohort may be related to these recommendations, albeit the referral rate was significantly higher among those with than without recorded formal diagnoses of overweight or obesity.

Substantial evidence supports concurrent treatment of obesity and comorbidities to achieve weight loss, avoid further weight gain, and improve obesity-related comorbidities. Therefore, it may be helpful for pediatricians to include the diagnosis of obesity in a patient’s problem list, to heighten awareness and remind providers to address weight concerns at subsequent clinic encounters.^22,23^ Notably, in a large adult study, documentation of an obesity diagnosis on a problem list was independently predictive of at least 5% weight loss.^22^

Comorbidity evaluation may confer benefits for improved weight outcomes. In adult studies, identifying obesity-related comorbidities has been described as a motivating factor to address weight concerns.^24^ The ACTION study found that people diagnosed with obesity were more likely to report successful weight loss.^25^ This supports previous findings that talking to patients about weight increases their desire to lose weight and enhances perceptions of weight issues.^26^ Evidence in pediatrics of the subject at issue is sparse and inconsistent. Among 4000 youth aged 10-18 years with overweight or obesity in a primary care network, BMI-z slopes per year decreased after HbA1c measurements. Among those who had an HbA1c test, the decline in BMI-z slope was greater among those with HbA1c in the prediabetes-range.^27^ However, another study did not find a positive effect on BMI change following cholesterol evaluation.^28^

Children with overweight and obesity may also benefit from health behavior and lifestyle treatment, which is child-focused, family-centered, or a coordinated approach to care. Interventions may involve pediatricians and other pediatric healthcare providers (dietitians, psychologists, nurses, exercise specialists, and social workers) and family members.^29^ We found among children with versus without recorded formal diagnoses of overweight and obesity, higher rates of referral for dietitian counseling and for endocrine evaluation, and a higher rate of subsequent BMI measurements. Gentile et al. ^30^ reported that implementing a point-of-care clinical reminder tool for diagnosing obesity was associated with improvement in documentation of diagnoses of obesity and counseling for nutrition and physical activity. Similarly, Thaker et al. ^31^ showed that documentation of obesity in the chart improved nutritional and physical activity counseling. However, Shaikh et al. ^32^ found that passive changes, such as automatic calculation of BMI, were insufficient to result in systematic improvements in assessment of weight and counseling for nutrition and physical activity.

Finally, in recent years, several anti-obesity medications were approved for use in the pediatric age group.^33^ Among the older age group of our cohort, among those with compared to those without recorded formal diagnoses of overweight and obesity, the rate was higher of prescriptions of anti-obesity medications. This was especially observed among females. Hidirsah et al. reported a similar finding,^13^ which may be explained by more frequent reports of weight-related stigmatizing events among females than males.^34^ This may explain why more females than males seek obesity treatment.

A surprising observation from our study was the significantly lesser decrease in BMI-Z score during a 1-2-year interval among the children with than without a recorded diagnosis of overweight or obesity. This observation was evident in both sexes and across both age groups in our cohort and remained consistent even after adjustment for possible confounders. A possible explanation is the older age and the higher BMI-Z score at baseline among those with a recorded diagnosis of overweight or obesity, albeit the results were consistent also after adjustment to age and baseline BMI-Z score. Additionally, in our cohort, those with vs. those without recorded diagnoses of overweight and obesity had significantly higher height-z scores. This may explain the underdiagnosis of those without recorded formal diagnoses, as PCP may diagnose overweight/obesity based on general appearance and body size. We also speculate that for some children who did not have a recorded formal diagnosis of overweight or obesity, their PCP may have discussed the issue with them and their parents. This may have increased motivation to make lifestyle changes. However, despite the lesser decrease in BMI-Z score among those with recorded formal diagnoses, these children had higher rates of screening for obesity-related comorbidities. This is important for preventing delays in diagnosing and treating these chronic health conditions.

The strengths of our study include the large cohort of children from the largest health maintenance organization in Israel, the use of objective measurements of BMI, representation of a large age group, different ethnicities (both Arab and Jewish ethnicity), and diverse SEP clusters.

Yet, some limitations exist. First, is the possibility of recording improbable BMI measurements due to mistakes in documented height and weight, although we excluded from the analysis those with invalid measurements. Second, is the absence of data about familial cardio-metabolic risk factors, which may have influenced the referral of the children for screening of obesity-related comorbidities. Third, is that referral for some of the blood tests, liver ultrasound, and sleep studies may have been due to other indications, and not necessarily due to overweight or obesity. Finally, though information was lacking, referral for physical activity programs, and psychosocial counseling may have impacted the changes in BMI-Z score.

In conclusion, primary care pediatricians must be aware of the importance of recording in medical records of formal diagnoses of overweight and obesity. Such documentation may encourage the PCP to be proactive, including further evaluations, such as screening for obesity-related comorbidities, and the treatment of obesity as a chronic disease. Further studies may be required to evaluate if improved recording of overweight/obesity diagnoses translates to improved treatment outcomes in the pediatric ages.

## Data Availability

The datasets generated during and/or analyzed during the current study are available from the corresponding author on reasonable request.
